# A causal link between circulating leukocytes and three major urologic cancers: a mendelian randomization investigation

**DOI:** 10.3389/fgene.2024.1424119

**Published:** 2024-06-19

**Authors:** Yi Zhi-gang, Wang Han-dong

**Affiliations:** Department of Nephrology, Huangshi Aikang Hospital Affiliated to Hubei Polytechnic University, Huangshi, Hubei, China

**Keywords:** leukocyte, neutrophil, prostate cancer, mendelian randomization, GWAS, genome-wide association study

## Abstract

**Purpose:**

This study aimed to explore the influence of serum leukocytes on urologic cancers (UC) using observation-based investigations. In the present study, Mendelian randomization (MR) was employed to assess the link between leukocyte count (LC) and the risk of UC development.

**Methods:**

Five LC and three major UC patient prognoses were obtained for MR analysis from genome-wide association studies (GWAS). Furthermore, in order to evaluate reverse causality, bidirectional studies were conducted. Finally, a sensitivity analysis using multiple methods was carried out.

**Results:**

There was no significant correlation found in the genetic assessment of differential LC between the co-occurrence of bladder cancer (BCA) and renal cell carcinoma (RCC). Conversely, an individual 1-standard deviation (SD) rise in neutrophil count was strongly linked to a 9.3% elevation in prostate cancer (PCA) risk ([odd ratio]OR = 1.093, 95% [confidence interval]CI = 0.864–1.383, *p* = 0.002). Reverse MR analysis suggested that PCA was unlikely to cause changes in neutrophil count. Additional sensitivity studies revealed that the outcomes of all MR evaluations were similar, and there was no horizontal pleiotropy. Primary MR analysis using inverse-variance weighted (IVW) revealed that differential lymphocyte count significantly influenced RCC risk (OR = 1.162, 95%CI = 0.918–1.470, *p* = 0.001). Moreover, altered basophil count also affected BCA risk (OR = 1.249, 95% CI = 0.904–1.725, *p* = 0.018). Nonetheless, these causal associations were not significant in the sensitivity analysis.

**Conclusion:**

In summary, the results revealed that increased neutrophil counts represent a significant PCA risk factor. The current research indicates a significant relationship between immune cell activity and the cause of UC.

## Introduction

Urologic cancer (UC) includes renal cell cancer (RCC), bladder cancer (BCA), prostate cancer (PCA), and other types of cancer affecting the urinary system. The Global Cancer Statistics 2020 report indicates that the cancer mentioned above categories account for approximately 12.5% of all newly diagnosed cancer cases. Additionally, PCA is the second most common type of cancer among males (Sung et al., 2021). Radical resection is the standard treatment for early-stage UC. However, in patients with advanced cancers, survival becomes a challenge. Therefore, it is crucial to clarify the underlying causes of UC, precisely in order to develop novel and effective treatments for UC. Several studies have consistently found a significant link between UC and the immune system, which in turn regulates the growth and advancement of tumors (Bou-Dargham et al., 2020; Rhea et al., 2021; Kashima and Braun, 2023). It is well established that tumors manipulate host immune responses to evade the immune system. In particular, tumor cells release targeted cytokines that recruit and stimulate myeloid-derived suppressor cell (MDSCs) synthesis. They also generate transforming growth factor-beta (TGF-β) and interleukin 10 (IL-10), which abrogate T lymphocytes, macrophages, and dendritic cells, thereby generating an immunosuppressive tumor microenvironment (TME) (Li et al., 2021).

Due to its crucial importance in the context of UC, the leukocyte count (LC), which is a component of the immune system, has significant promise as a reliable bioindicator for UC. Leukocytes are commonly distributed throughout the human body, especially in regions that are specifically associated with the hematologic and lymphatic systems. Leukocytes have five primary subtypes: lymphocytes, monocytes, neutrophils, eosinophils, and basophils. Together, these cells strongly modulate tumor growth, as host immunity is highly reliant on an intricate balance between various immune cells. When an immunological response occurs, like in the case of cancer, LC is substantially affected. Previous studies have indicated that individuals with RCC often had elevated CD4/CD8 T-lymphocyte ratios, decreased dendritic cell counts, and elevated granulocyte contents in their blood.

Moreover, these alterations are associated with disease progression (Hase et al., 2011). There are other reports on the prognostic relevance of the neutrophil-to-lymphocyte ratio (NLR) in RCC (Ohno et al., 2010; Keizman et al., 2012; Ohno et al., 2012; Sejima et al., 2013). Neutrophils are known to regulate the inflammatory response, and prolonged inflammation significantly increases the probability of developing tumors. Bladder neutrophils in patients with bladder cancer who undergo post-surgical *bacillus* Calmette-Guerin (BCG) perfusion show signs of releasing an excessive amount of factors that stimulate apoptosis while also attracting factors from other immune cells. This indicates a significant involvement of neutrophils in the evolution of BCA (Simons et al., 2007).

Moreover, other studies have examined the correlation between the NLR and BCA. This study provided evidence that NLR is strongly correlated with a poorer prognosis in patients with BCA. However, no significant relationship was observed between NLR and the progression or recurrence of BCA (Albayrak et al., 2016).

Additionally, one investigation involving docetaxel chemotherapy-treated castration-resistant PCA patients revealed that reduced lymphocyte and elevated monocyte levels were strongly correlated with decreased overall and progression-free survival (Shigeta et al., 2016). A meta-analysis reported that monocyte and lymphocyte concentrations are critically linked to patient prognosis among PCA patients (Peng and Luo, 2019). Nonetheless, one Swedish study revealed no direct association between LC and PCA risks. It is important to note that the study, as mentioned above, examined LC among men aged 45–55 years, among whom LC increased the PCA risk by 44% (Julin et al., 2015). Currently, it remains uncertain if there is a genetic association between LC and the development of urologic tumors. This uncertainty is mainly because of the limited number of participants in previous studies and the possibility of other factors influencing the results in observational studies.

Mendelian randomization (MR) is commonly adopted for etiological inferences in genetic epidemiological research (Sekula et al., 2016). In recent times, MR has also been employed to predict pathogenic relationships between two complex disorders (Emdin et al., 2017). The objective of this study was to use MR analysis to examine the genetic connection between LC and UC by evaluating the Genome-Wide Association Study (GWAS)-based LC and UC data. The current findings and conclusions offer innovative avenues for the prevention and diagnosis of UC.

## Methods

### Study design

**FIGURE 1:**
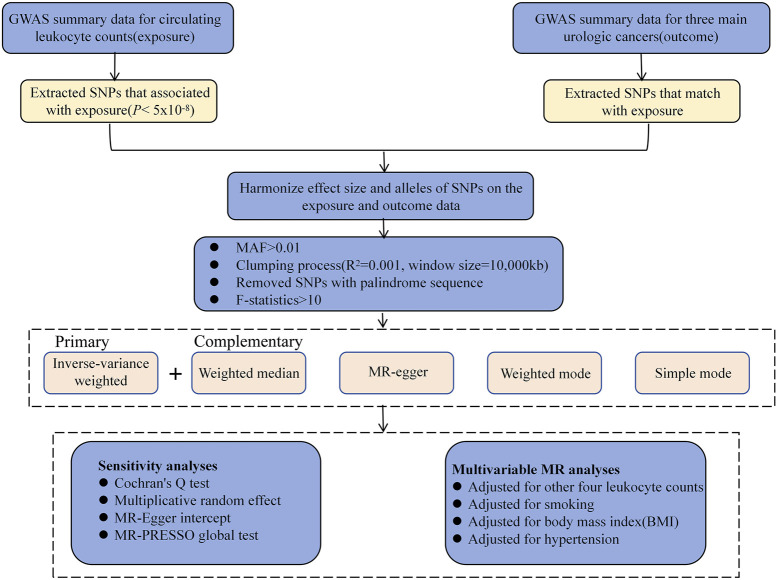
Study design and overview of our Mendelian randomization (MR) study. GWAS, genome-wide association studies; MAF, minor allele frequency; SNP, single nucleotide polymorphism.

Using publicly available data from the GWAS database, a two-sample MR analysis was performed to assess the causal relationship of serum LC (lymphocytes, monocytes, neutrophils, eosinophils, and basophils) and three UC patient prognoses (RCC, BCA, and PCA) (Figure 1). For our analysis, three major assumptions were made: (1) there is a strong correlation between genetic variables and exposure, (2) variable-outcome modulation is adjusted by exposure, and (3) variables-outcome modulation is not modulated by exposure and confounding factors (Lawlor et al., 2008). All research protocols adhered to the World Medical Association’s Declaration of Helsinki. Institutional ethical approval was waived because data were retrieved from the publicly available GWAS database, and all information was collected with ethical consent from the appropriate institutions. All of the presented analyses were conducted using summary-level data.

### Data source

Summary statistics were downloaded from GWAS, a database under the Blood Cell Consortium (BCC). BCC Phase 2 comprised 563,946 European subjects from 26 GWAS cohorts. Patients with blood malignancy, acute medical/surgical illness, myelodysplastic syndrome, bone marrow transplant, congenital/hereditary anemia, human immunodeficiency virus (HIV), end-stage kidney disease, splenectomy, cirrhosis, or extreme blood cell counts were excluded from the study. Supplementary Table S1 provides a concise description of our data sources, with additional information available from the original research (Chen et al., 2020).

All available UC data from the Public Integrative Epidemiology Unit (IEU) GWAS database (https://gwas.mrcieu.ac.uk/) were examined in order to evaluate the relationship between LC and UC risk systematically. The GWAS with the biggest sample populations was selected, and seven GWAS with summary data for various UC types were eventually collected before analysis. The largest GWAS, which included the most freely available data on all three primary UC types—RCC, BCA, and PCA—was selected for analysis (Supplementary Table S1).

### Screening of instrumental variables (IVs) for serum LC

As genetic tools, the single nucleotide polymorphisms (SNPs) associated with the five major LCs that have a genome-wide significance of *p* < 5 × 10^−8^ were found. A clumping study was conducted to verify that SNPs are independent. The SNPs underwent pruning at a stringent linkage disequilibrium (LD) at *R* ^2^ < 0.001 within a 10,000-kb range. The variance proportions of the corresponding LC estimated by the selected SNPs and F-statistics were regarded as measures of instrumental strength (Palmer et al., 2012). The F-value for all IVs was adjusted to >10 to ensure a weak bias of <10% for a minimum of 95% of the time (Supplementary Table S2).

### Statistical analysis

MR analyses were conducted using five distinct statistical methods. Initially, a primary Two-sample MR analysis using the inverse-variance weight (IVW) approach was performed to measure the causal association between LC (lymphocytes, monocytes, neutrophils, eosinophils, and basophils) and the risk of three distinct UC types (Burgess et al., 2013). During this analysis, the coefficient ratio was computed to assess causal outcomes. In addition, MR-Egger regression was adopted to explore horizontal pleiotropy between the IVs and the three UC categories. The weighted median method (WM) required only half of the relevant SNPs to supplement the IVW analysis (Bowden et al., 2016). Furthermore, the weighted and simple mode analyses were employed to estimate causal outcomes.

Utilizing Cochran’s Q, SNP heterogeneity was evaluated. Interestingly, since a random-effect model for the IVW technique was used, heterogeneity did not affect MR-based predictions. Using intercept as a horizontal pleiotropy indicator, MR-Egger regression was used to investigate potential horizontal pleiotropy influencing the three primary UC types through additional physiological networks. An innovative MR method known as MR-pleiotropy residual sum and outlier (MR-PRESSO) is a variation of the IVW method. The MR-PRESSO global test was used for the overall horizontal pleiotropy assessment. Upon pleiotropy detection (*p* < 0.05), the MR-PRESSO outlier test was employed to identify discrete pleiotropic outliers by computing the square residual sums (Verbanck et al., 2018). Finally, the remaining genetic variations were subjected to causal prediction using the IVW technique after outlier reduction.

A variation of the traditional MR methodology referred to as multivariable MR (MVMR) includes several related exposures into account in a single model, making it easier to identify the independent correlation between a single exposure and an outcome. In this study, an MVMR was carried out with potential SNP correlations such as smoking (Kumar et al., 2023), hypertension (Campi et al., 2023), and body mass index (BMI) (Liu et al., 2018) as covariates to elucidate the positive influences of LC, independent of UC-related risk factors. After adjusting for the effects of the remaining four leukocyte subtypes, we then used MVMR to explain the role of individual leukocyte subtypes on UC, taking into account the direct modulation between leukocyte subtypes.

A reverse-direction MR analysis was performed to evaluate whether there is genetic evidence for the possibility that UC alters circulating LC. A stringent statistical threshold (*p* < 5 × 10^−8^) was used to select genome-wide significant SNPs for UC. In this reverse-direction analysis, IVW, MR-Egger and weighted median analyses were performed as described above.

The data is presented as the mean outcome per 1 standard deviation (SD) rise in the genetically predicted LC and UC levels, collectively with their corresponding 95% confidence intervals (CIs). TwoSampleMR and MR-PRESSO packages in R (version 4.0.3) were used for all data analysis. Statistical significance was set at *p* < 0.05.

## Results

### The causal association between serum LC and RCC

**FIGURE 2:**
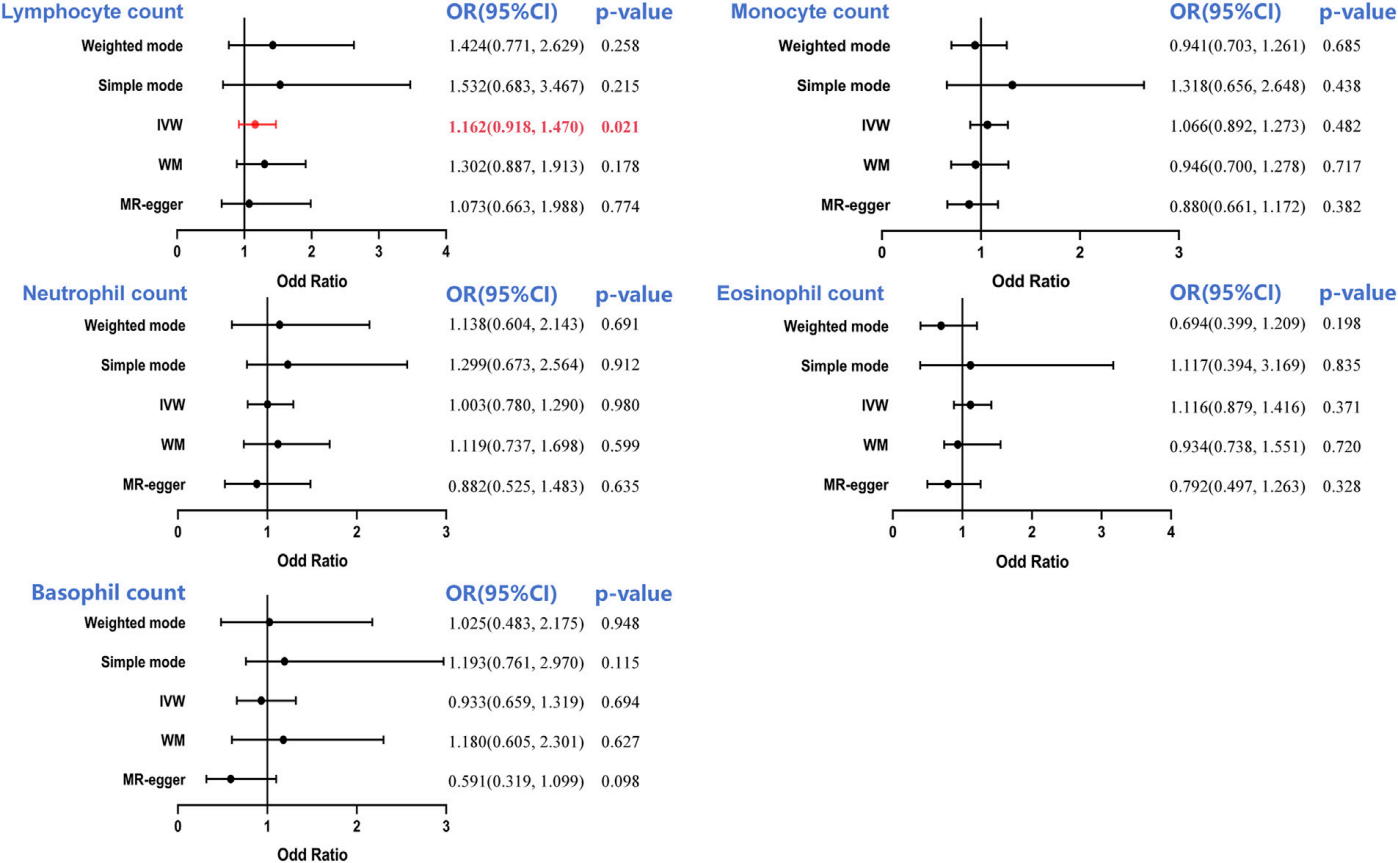
Forest plot to visualize the causal effect of circulating leukocyte counts on the risk of renal cell cancer. IVW, inverse-variance weighted; OR, odd ratio; CI, confidence interval. Statistical significance was defined as *p* < 0.05.

The main IVW-based prediction for the five leukocyte subtypes was that increased RCC risk was causally correlated with a genetically predicted 1 SD increase in LC ([odd ratio]OR = 1.162, 95%CI = 0.918–1.470, *p* = 0.001) (Supplementary Figure S1A). Nonetheless, no causal link in the monocyte (OR = 1.066, 95%CI = 0.892–1.273, *p* = 0.482), neutrophil (OR = 1.003, 95%CI = 0.780–1.290, *p* = 0.980), eosinophil (OR = 1.116, 95%CI = 0.879–1.416, *p* = 0.371) and basophil counts (OR = 0.933, 95%CI = 0.659–1.319, *p* = 0.4694) was observed (Figure 2).

Based on Cochran’s Q statistic, this study similarly found no evidence of significant heterogeneity in the counts of monocytes, neutrophils, and basophils (all *p* > 0.05, Table 1). Additional sensitivity evaluation revealed slight heterogeneity in lymphocyte (*p* = 0.013) and eosinophil counts (*p* = 0.004). However, IVW model usage involving multiplicative random effects failed to change the results (lymphocyte count: OR = 1.162, *p* = 0.022; eosinophil count: OR = 1.115, *p* = 0.369). Similarly, the MR-Egger intercept test *p*-value and the MR-PRESSO global pleiotropy test *p*-value showed that pleiotropy-based bias was not present in the IVW analysis (all *p* > 0.05, Table 1; Supplementary Figure S1B).

**TABLE 1:** Sensitivity MR analyses evaluating the causal effects of circulating leukocyte counts on three main urologic cancers.

Exposure	Outcomes	n (SNPs)	Heterogeneity test		Pleiotropy test	
			Cochran’s Q test (*p*-value)	Multiplicative random effect (*p*-value)	MR-egger intercept (*p*-value)	MR-PRESSO global test (*p*-value)
Lymphocyte count	Renal cell cancer	422	488.26 (0.013)	1.162 (0.022)	2.22 × 10^−3^ (0.712)	0.654
	Bladder cancer	422	417.98 (0.532)	N/A	−8.53 × 10^−3^ (0.101)	0.120
	Prostate cancer	441	1141.88 (8.51 × 10^−64^)	1.028 (0.378)	3.58 × 10^−4^ (0.819)	0.083
Monocyte count	Renal cell cancer	422	417.63 (0.537)	N/A	7.35 × 10^−3^ (0.095)	0.311
	Bladder cancer	422	440.07 (0.251)	N/A	1.61 × 10^−3^ (0.701)	0.765
	Prostate cancer	441	1073.41 (8.51 × 10^−55^)	1.012 (0.642)	1.19 × 10^−3^ (0.329)	0.891
Neutrophil count	Renal cell cancer	354	367.85 (0.282)	N/A	3.41 × 10^−3^ (0.578)	0.076
	Bladder cancer	354	370.48 (0.251)	N/A	1.47 × 10^−3^ (0.798)	0.668
	Prostate cancer	368	1264.47 (6.03 × 10^−99^)	1.051 (0.013)	3.07 × 10^−4^ (0.874)	0.145
Eosinophil count	Renal cell cancer	375	449.74 (0.004)	1.115 (0.369)	0.011 (0.095)	0.332
	Bladder cancer	375	421.34 (0.042)	1.089 (0.438)	6.34 × 10^−3^ (0.273)	0.617
	Prostate cancer	388	980.42 (3.36 × 10^−53^)	1.048 (0.123)	1.19 × 10^−3^ (0.456)	0.058
Basophil count	Renal cell cancer	162	159.41 (0.498)	N/A	0.014 (0.084)	0.431
	Bladder cancer	162	159.85 (0.511)	N/A	1.56 × 10^−3^ (0.832)	0.399
	Prostate cancer	168	417.72 (1.83 × 10^−23^)	1.081 (0.134)	6.26 × 10^−4^ (0.793)	0.468

Cochran’s Q test was derived from the IVW, estimates and used to explore potential heterogeneity between IVs. When significant heterogeneity (*p* < 0.05) was detected, the IVW, model of multiplicative random effects were used to detect it. The MR-Egger regression-derived MR-Egger intercept and MR-PRESSO, global test were used to examine directional pleiotropy of IVs, with *p* < 0.05 as the threshold for significant pleiotropy.

### Causal correlation between serum LC and BCA

**FIGURE 3:**
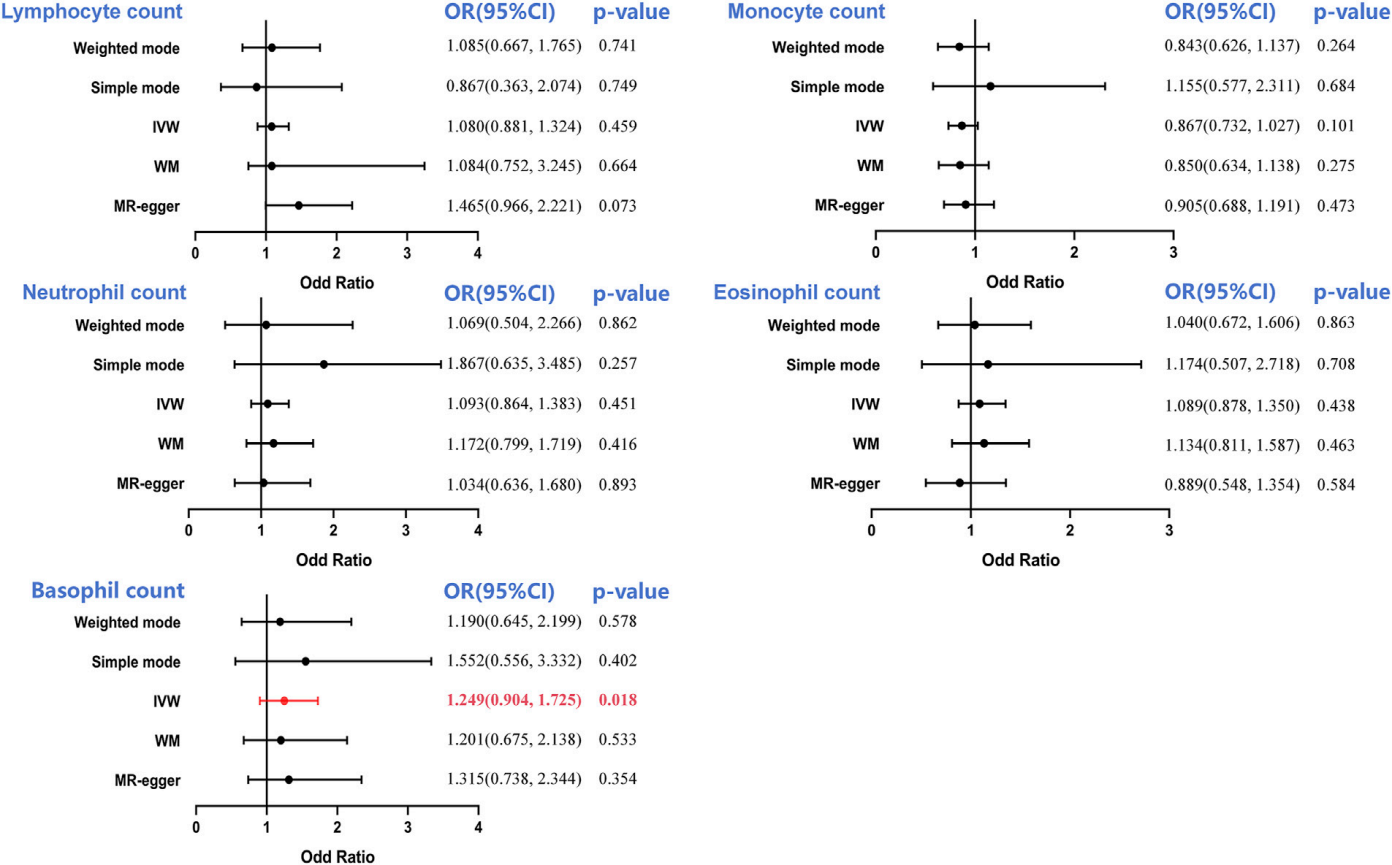
Forest plot to visualize the causal effect of circulating leukocyte counts on the risk of bladder cancer. IVW, inverse-variance weighted; OR, odd ratio; CI, confidence interval. Statistical significance was defined as *p* < 0.05.

Strong evidence suggested a causal association between lymphocyte (OR = 1.080, 95%CI = 0.881–1.324, *p* = 0.459), monocyte (OR = 0.867, 95%CI = 0.732–1.027 *p* = 0.101), neutrophil (OR = 1.096, 95%CI = 0.864–1.383, *p* = 0.451), eosinophil counts (OR = 1.089, 95%CI = 0.878–1.350, *p* = 0.438), and overall BCA occurrence, as was evidenced by IVW analysis (Figure 3). However, the findings showed that basophil count regulated BCA risk. A 1-SD rise in basophil count was shown to be closely associated with a 24.9% increase in BCA risk, according to our genetic estimation (OR = 1.249, 95% CI = 0.904–1.725, *p* = 0.018) (Figure 3; Supplementary Figure S1C).

The Cochran’s Q test was utilized to determine pleiotropy, and the results showed that there was no significant heterogeneity between the genetic instruments, with *p*-values of 0.532 for lymphocyte count, 0.251 for monocyte count, 0.251 for neutrophil count, and 0.511 for basophil count. A slight heterogeneity in the eosinophil count was observed (*p* = 3.36 × 10^−53^). However, IVW model usage involving multiplicative random effects failed to change the results (OR = 1.048, *p* = 0.123). Similarly, there was no evidence of bias associated with directional pleiotropy in any of these MR-Egger studies with intercepts close to 0 and all *p* values more than 0.05. The results indicated above were confirmed by the *p*-value of the MR-PRESSO global pleiotropy test (Table 1; Supplementary Figure S1D).

## The causal association between serum LC and PCA

**FIGURE 4:**
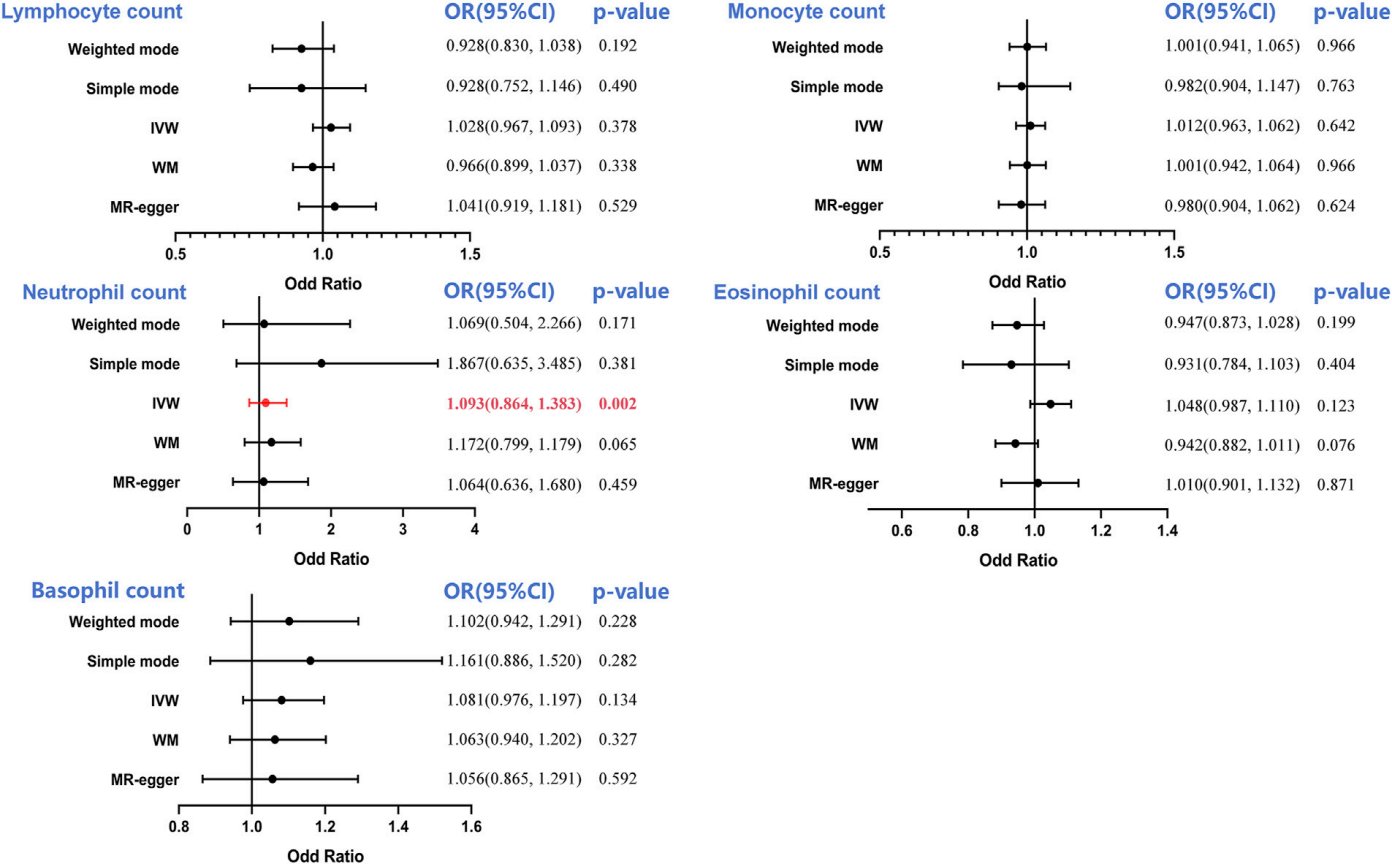
Forest plot to visualize the causal effect of circulating leukocyte counts on the risk of prostate cancer. IVW, inverse-variance weighted; OR, odd ratio; CI, confidence interval. Statistical significance was defined as *p* < 0.05.

It has been shown that the IVW technique produced notable significance (OR = 1.093, 95%CI = 0.864–1.383, *p* = 0.002) using MR analysis of the neutrophil count and PCA, suggesting that an increase in the neutrophil count constituted a strong risk factor for PCA (Supplementary Figure S1E). Interestingly, WM, MR-Egger, simple mode, and weighted mode analyses did not generate the same association between neutrophil count and PCA; however, the trend was the same. There was no causal influence on the lymphocyte (OR = 1.028, 95%CI = 0.967–1.093, *p* = 0.378), monocyte (OR = 1.012, 95%CI = 0.963–1.062, *p* = 0.642), eosinophil (OR = 1.048, 95%CI = 0.987–1.110, *p* = 0.123) and basophil counts (OR = 1.081, 95%CI = 0.976–1.197, *p* = 0.134) observed in this study (Figure 4).

Following this, a sensitivity analysis was performed on each of the five exposure-outcome association categories. According to Cochran’s Q test, every association had potential heterogeneity (all *p* > 0.05, Table 1; Supplementary Figure S1F). Thus, the random-effects IVW approach was adopted to minimize this influence. The IVW model involving multiplicative random effects indicated a strong causal impact of the neutrophil count on PCA (OR = 1.051, *p* = 0.013). No pleiotropy was evident in the corresponding MR-Egger intercept (all *p* > 0.05) or MR-PRESSO global pleiotropy test (all *p* > 0.05).

### MVMR analyses of positive outcomes

**FIGURE 5:**
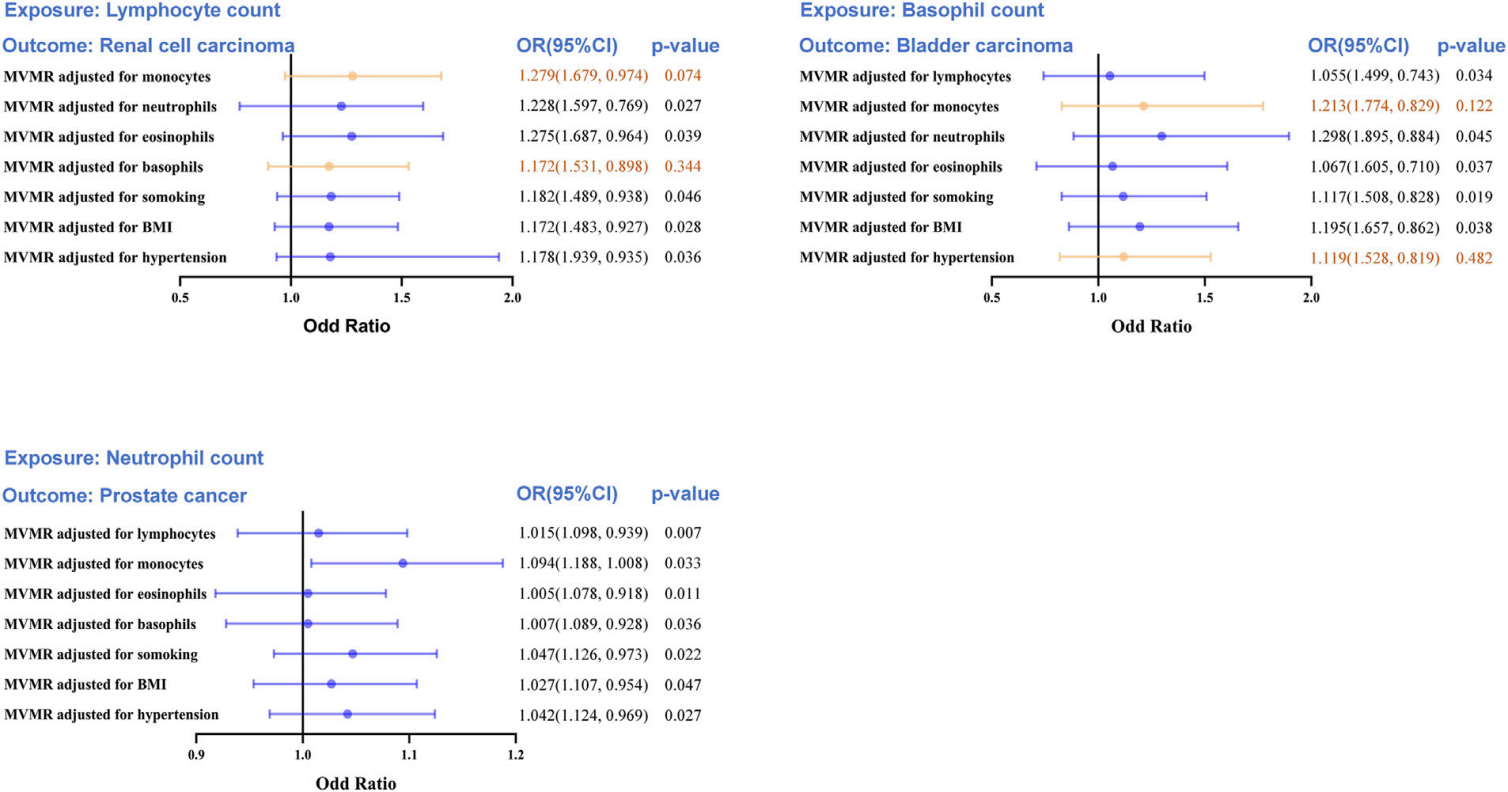
Multivariable MR (MVMR) analyses to assess the positive causal associations between leukocyte counts and risk of urologic cancers. MVMR, multivariable Mendelian randomization; OR, odd ratio; CI, confidence interval. Statistical significance was defined as *p* < 0.05.

Strong causal relationships between neutrophil count-PCA, basophil count-BCA, and lymphocyte count-RCC were found in earlier two-sample MR analyses. An MVMR analysis for the three aforementioned causal correlations was performed in order to eliminate interference from the leukocyte subtype and common confounders (such as smoking, hypertension, and BMI) (Figure 5).

The MVMR analysis also validated the causal association between lymphocyte count and RCC following UC risk factor adjustment (smoking, hypertension, and BMI), as well as the impact of the remaining two leukocyte subtypes (neutrophil and eosinophil counts). When considering monocyte (OR = 1.279, 95%CI = 0.974–1.679, *p* = 0.0074) and basophil counts (OR = 1.172, 95%CI = 0.898–1.531, *p* = 0.344), the direct lymphocyte count-based influence on RCC was abolished entirely. Similarly, the basophil count-based influence on BCA was eliminated when taking into account monocyte count (OR = 1.213, 95%CI = 0.829–1.774, *p* = 0.122) and hypertension (OR = 1.119, 95%CI = 0.819–1.528, *p* = 0.482). Collectively, these results indicate a shortage of compelling data substantiating a cause-and-effect connection between LC and RCC or BCA. The MVMR analysis revealed a clear causal connection between neutrophil count and PCA risk, even after accounting for the other four leukocyte subtypes and risk factors of UC (smoking, hypertension, and BMI).

### Reverse MR analysis to assess the effect of UC on LC

In order to investigate the possibility that reverse causation may be the cause of our results, a thorough reverse MR analysis was conducted where the exposure was the risk of UC, and the outcome was the counts of the LC. While RCC, BCA and PCA were all found to have an impact on the differential leukocyte subtypes in the IVW analysis, the practical significance of these findings is highly debatable due to the extremely small effect sizes (Supplementary Table S3). Furthermore, in both the weighted median and MR-Egger analyses, these causal effects failed to reach statistical significance. Therefore, there was insufficient evidence to support any inverse relationships. In particular, no causal correlations between PCA and neutrophil count were discovered using the IVW technique (OR = 1.001, 95%CI = 0.996–1.006, *p* = 0.110) (Supplementary Table S3). Weighted median and MR-Egger analyses produced comparable findings.

## Discussion

Large-scale publicly available genomic datasets were subjected to an MR analysis in the current work in order to identify any potential causal relationships between LC and three different UC categories. The primary finding was that genetically determined higher neutrophil levels increased PCA risk. On the other hand, there was no evidence that the number of monocytes or lymphocytes and the risk of UC were related. The special value of this analysis is that, in spite of the scarcity of observational studies on the relationships between LC and various UC types, the current research thoroughly evaluated the association between individual differential LC and the three different UC types. Furthermore, the MR approach was used to reduce bias due to confounding factors and reverse causality; as a result, conclusions on causal associations can be made rather than only making assumptions.

Diversity is an integral feature of the immune system and strictly modulates an individual’s risk of contracting immune-related diseases. Although serum immune cell contents are likely to change under infection or injury, their levels are highly variable, even among “healthy” individuals (Patin et al., 2018). Additionally, there are reports that immune cell composition is intricately linked to cancer risk (Le Cornet et al., 2020) in healthy people without prior corresponding diseases. Nevertheless, the precise correlation between these two factors remains uncertain. The current study investigated the UC data obtained from the Blood Cell Consortium, where the LC was within the normal range (Chen et al., 2020). Based on the extensive data provided in this study, LC has the potential to serve as a bioindicator for assessing the risk of UC in patients without any disease.

Recent research indicates that the growth and advancement of tumors have a strong relationship to the inflammatory response and contact with the tumor microenvironment (TME) (Morimoto et al., 2014; Chen et al., 2017). Aberrant tumor growth often promotes the release of pro-inflammatory factors from surrounding cells and invading immune cells further influence tumor proliferation and angiogenesis via cytokine release, which ultimately controls tumor metastasis and progression. Elevated neutrophil counts in the blood of individuals with malignant tumors are independent predictors of unfavorable clinical outcomes (Najmeh et al., 2017). Neutrophils and other inflammatory mediators release cytokines to generate a conducive environment for tumor growth and reproduction. Furthermore, neutrophils release vascular endothelial growth factor, which assists in new blood vessel formation while encouraging tumor growth and invasion (Cools-Lartigue et al., 2013). Monocytes, another class of relevant immune cells, also accelerate tumor growth and metastasis by differentiating into tumor-associated macrophages. Lymphocytes, a critical constituent of tumor-specific immunity, aid in the destruction and apoptosis of tumor cells. The NLR can effectively depict the inflammatory and immunological condition of patients with cancers, considering its significant involvement in tumor biology. Recent reports have confirmed the strong predictive ability of NLR in breast, gastric, lung, and other types of cancer (Chen et al., 2019; Hirahara et al., 2019; Kang et al., 2019).

Nevertheless, there is currently no agreement on the role of PCA diagnosis. This study has shown a strong and independent causal relationship between the number of neutrophils in the blood serum and the risk of PCA. The present results provide strong support for the findings published by Kwon et al., who concluded that an increased neutrophil status in the early stages of the disease is closely associated with the development of aggressive PCA (Kwon et al., 2016).

Furthermore, an increased risk of PCA development has been associated with elevated neutrophil counts in individuals with various infectious diseases, including chronic prostatitis (Sfanos and De Marzo, 2012). Neutrophil-based cytokines and proteins have also been examined for their ability to indicate tumor progression and severity in various cancer types (Donskov, 2013). Currently, there have been no documented findings on the possible correlation between the four categories of leukocytes and the risk of prostate cancer. This could be due to the fact that any factor or microenvironment that promotes tumor growth also enhances the production of neutrophils. Additionally, the cytokines and inflammatory mediators produced by neutrophils enhance TME formation and encourage vascular epithelial cell growth factor (VEGF) synthesis and release, which in turn fortifies tumor angiogenesis and progression (Massena et al., 2015; Zhu et al., 2020). In conclusion, these findings offered compelling evidence of neutrophil involvement in PCA pathogenesis. This study also underlined the potential of the neutrophil count as a bioindicator for PCA risk assessment.

RCC and BCA are the two remaining forms of UC. Previous observational investigations have reported a strong association between the diseases mentioned above and serum LC (Guan et al., 2021; Gao et al., 2022). Moreover, *in vivo,* examinations validated the speculation that LC enhances tumor progression and metastasis in RCC mice via the secretion of excessive chemokine (C-C motif) ligand 18 (CCL18) and TGF-β1. Additionally, inflammation attracts monocytes, macrophages, and neutrophils, which produce reactive oxygen and nitrogen species, ultimately damaging tissues, proteins, lipids, and DNA. These cellular and molecular changes result in tumorigenesis and angiogenesis, as is evident in BCA (Xu et al., 2002). However, there was no discernible substantial association between genetically estimated LC and RCC or BCA. In particular, a strong causal relationship between basophils and BCA and lymphocyte count and RCC has been shown using two-sample MR.

Nevertheless, these correlations vanished when common confounding variables and the remaining four leukocyte subtypes were taken into account during MVMR analysis. Based on the various sensitivity studies and the conflicting predictions, it can be inferred that genetically determined LC does not have an impact on RCC or BCA. A potential explanation for the difference between previous and present findings is that the earlier MR investigation investigated the effects of long-term increased exposure to LC on the risk of developing UC (Davies et al., 2018). In contrast, observational investigations generally have limited follow-up evaluations and may only reveal the short-term effects of LC on UC risk.

Genetic correlations confirm a cause-and-effect connection, indicating that variations in gene expression lead to varied observable traits. Therefore, in MA analysis, the genotype is included as IV in order to reduce the occurrence of reverse causality, a trend frequently observed in randomized controlled trials. Conversely, the MR analysis remained unaffected by social and behavioral aspects. This work utilized MR analysis of large-scale GWAS summary data to obtain consistent findings. The current sensitivity study successfully addressed the issues of horizontal pleiotropy and heterogeneity, ensuring the reliability of the conclusions. This study additionally performed MVMR analysis to validate the neutrophil count as an independent risk factor for PCA. It is crucial to acknowledge that the current research has specific constraints. Initially, the data about the exposure and outcome were obtained from European populations. Therefore, these findings may not be applicable to different populations. The leukocyte subtype counts were only available once per sample.

Therefore, it was hypothesized that the sample LCs remained consistent. This analysis failed to represent the actual characteristics of the samples accurately, and it also prevented us from studying the impact of significant changes in the data within a brief timeframe. Due to the limitations in collecting data, the relationship between LC trends and the risk of developing and progressing UC was not studied. Second, MR analysis does not permanently eliminate pleiotropic effects. Nonetheless, the observed effect estimates were consistent across numerous sensitivity analyses, indicating low confounding and bias. Finally, MR analysis basically investigates causal relationships; the underlying physiological or pathological process requires further studies.

## Conclusion

In conclusion, the present study employed MR analysis to eliminate reverse causality- and socio-physiological factor-related biases and demonstrated that an augmented neutrophil count strongly enhances PCA risk. Neutrophil count, as a subtype of leukocytes, is both efficient and easily detectable, making it a possible target for therapeutic intervention in PCA. Additional research is necessary to clarify the underlying signaling mechanism that connects LC and UC, as well as to evaluate the efficacy of neutrophil count as a target for diagnosing and treating PCA.

## Data Availability

The original contributions presented in the study are included in the article/Supplementary Material, further inquiries can be directed to the corresponding author.

## Supplementary material

SUPPLEMENTARY FIGURE S1 (A-B) scatter plot and funnel plot of lymphocyte count on RCC; (C-D) scatter plot and funnel plot of basophil count on BCA; (E-F) scatter plot and funnel plot of neutrophil count on PCA
