# Supplementary material for: A causal link between circulating leukocytes and three major urologic cancers: a mendelian randomization investigation
Source: Front Genet. 2024 Jun 19;15:1424119. doi: 10.3389/fgene.2024.1424119 (PMC11220253; doi:10.3389/fgene.2024.1424119)
Supplement: Supplementary file 1 [file Table1.docx]

**Table S1. Characteristics of the cohorts included in the MR study.**

| **Phenotype** | **Description** | **Consortium/Study** | **Unit** | **Sample size** |
| --- | --- | --- | --- | --- |
| **Exposure data** |  |  |  |  |
| Lymphocyte count | Aggregate count of lymphoid cells per unit volume of blood | Blood Cell Consortium | per nL | 563,946 |
| Monocyte count | Count of monocytes per unit volume of blood | Blood Cell Consortium | per nL | 563,946 |
| Neutrophil count | Count of neutrophils per unit volume of blood | Blood Cell Consortium | per nL | 563,946 |
| Eosinophil count | Count of eosinophils per unit volume of blood | Blood Cell Consortium | per nL | 563,946 |
| Basophil count | Count of basophils per unit volume of blood | Blood Cell Consortium | per nL | 563,946 |
|  |  |  |  |  |
| **Outcome data** |  |  |  |  |
| Renal cell cancer | Malignant neoplasm of kidney, except renal pelvis | FinnGen | logOR | 218,792 |
| Bladder cancer | Malignant neoplasm of bladder | FinnGen | logOR | 218,792 |
| Prostate cancer | GWAS analyses of more than 140,000 men identify 63 new prostate cancer susceptibility loci | PRACTICAL | logOR | 140,254 |

| **Phenotype** | **Cases/controls** | **Ancestry** | **IEU ID** | **Summary data URL** | **PMID** | **URL for detailed phenotype description** |
| --- | --- | --- | --- | --- | --- | --- |
| **Exposure data** |  |  |  |  |  |  |
| Lymphocyte count | 151,807/412,139 | European | ieu-b-32 | https://gwas.mrcieu.ac.uk/datasets/ieu-b-32/ | PMID: 32888493 | https://pubmed.ncbi.nlm.nih.gov/32888493/ |
| Monocyte count | 151,807/412,139 | European | ieu-b-31 | https://gwas.mrcieu.ac.uk/datasets/ieu-b-31/ | PMID: 32888493 | https://pubmed.ncbi.nlm.nih.gov/32888493/ |
| Neutrophil count | 151,807/412,139 | European | ieu-b-34 | https://gwas.mrcieu.ac.uk/datasets/ieu-b-34/ | PMID: 32888493 | https://pubmed.ncbi.nlm.nih.gov/32888493/ |
| Eosinophil count | 151,807/412,139 | European | ieu-b-33 | https://gwas.mrcieu.ac.uk/datasets/ieu-b-33/ | PMID: 32888493 | https://pubmed.ncbi.nlm.nih.gov/32888493/ |
| Basophil count | 151,807/412,139 | European | ieu-b-29 | https://gwas.mrcieu.ac.uk/datasets/ieu-b-29/ | PMID: 32888493 | https://pubmed.ncbi.nlm.nih.gov/32888493/ |
|  |  |  |  |  |  |  |
| **Outcome data** |  |  |  |  |  |  |
| Renal cell cancer | 971/217,821 | European | finn-b-C3_KIDNEY_NOTRENALPELVIS | https://gwas.mrcieu.ac.uk/datasets/finn-b-C3_KIDNEY_NOTRENALPELVIS/ | N/A | https://r8.finngen.fi/pheno/C3_KIDNEY_NOTRENALPELVIS_EXALLC |
| Bladder cancer | 111,5/217,677 | European | finn-b-C3_BLADDER | https://gwas.mrcieu.ac.uk/datasets/finn-b-C3_BLADDER/ | N/A | https://r8.finngen.fi/pheno/C3_BLADDER_EXALLC |
| Prostate cancer | 79,148/61,106 | European | ieu-b-85 | https://gwas.mrcieu.ac.uk/datasets/ieu-b-85/ | PMID: 29892016 | https://pubmed.ncbi.nlm.nih.gov/29892016/ |
