# Supplementary material for: A causal link between circulating leukocytes and three major urologic cancers: a mendelian randomization investigation
Source: Front Genet. 2024 Jun 19;15:1424119. doi: 10.3389/fgene.2024.1424119 (PMC11220253; doi:10.3389/fgene.2024.1424119)
Supplement: Supplementary file 2 [file Table3.docx]

**Table S3. Reverse MR analyses evaluating the causal effects of three major urologic cancers on leukocyte counts**

| **Exposure** | **Outcome** | **IVW** | | **MR-Egger** | | | | **Weighted median** | |
| --- | --- | --- | --- | --- | --- | --- | --- | --- | --- |
|  |  | OR (95% CI) | *P* | OR (95% CI) | *P* | Intercept | Intercept *P* | OR (95% CI) | *P* |
| **RCC** | Lymphocyte count | 1.001 (0.999-1.003) | 0.548 | 1.028(0.967- 1.093) | 0.661 | 0001 | 0.997 | 1.001 (0.998-1.002) | 0.176 |
|  | Monocyte count | 1.001 (0.995-1.007) | 0.954 | 1.001(0.942- 1.064) | 0.092 | -0.001 | 0.815 | 1.002 (0.997-1.006) | 0.604 |
|  | Neutrophil count | 1.006 (0.994-1.018) | 0.138 | 1.012(0.963- 1.062) | 0.841 | -0.003 | 0.292 | 1.001 (0.998-1.004) | **0.012** |
|  | Eosinophil count | 1.003 (0.998-1.008) | **0.003** | 0.928(0.833- 1.038) | 0.857 | -0.016 | 0.526 | 1.002 (0.997-1.006) | 0.439 |
|  | Basophil count | 1.002 (0.998-1.007) | **0.026** | 1.172(0.799- 1.179) | 0.300 | -0.002 | 0.216 | 1.003 (0.998-1.008) | 0.444 |
|  |  |  |  |  |  |  |  |  |  |
| **BCA** | Lymphocyte count | 1.007 (0.957-1.061) | 0.359 | 1.172(0.799- 1.719) | 0.649 | 0.363 | 0.391 | 1.042 (0.835-1.301) | 0.184 |
|  | Monocyte count | 0.999 (0.997-1.002) | 0.648 | 1.010(0.900- 1.132) | 0.557 | 0.016 | 0.800 | 1.117(1.508-0.828) | 0.463 |
|  | Neutrophil count | 0.997 (0.988-1.006) | 0.366 | 1.093(0.864- 1.383) | 0.439 | -0.002 | 0.312 | 1.195(1.657-0.862) | 0.633 |
|  | Eosinophil count | 0.992 (0.954-1.032) | 0.841 | 1.048(0.987- 1.119) | 0.341 | 0.003 | 0.069 | 1.119(1.528-0.819) | 0.700 |
|  | Basophil count | 1.004 (1.001-1.006) | **0.004** | 1.001 (0.997-1.005) | 0.694 | -0.003 | 0.526 | 1.002 (0.997-1.006) | 0.863 |
|  |  |  |  |  |  |  |  |  |  |
| **PCA** | Lymphocyte count | 1.002 (1.000-1.005) | 0.663 | 1.001 (0.997-1.005) | 0.667 | -0.003 | 0.228 | 0.591(0.319-1.099) | 0.624 |
|  | Monocyte count | 1.001 (0.991-1.01) | **0.014** | 1.034(0.636-1.680) | 0.131 | -0.010 | 0.134 | 1.181(0.605- 2.313) | 0.966 |
|  | Neutrophil count | 1.001 (0.996-1.006) | 0.110 | 1.004 (0.996-1.012) | 0.579 | 0.529 | 0.327 | 1.033(0.659-1.519) | 0.642 |
|  | Eosinophil count | 0.999 (0.998-1.001) | 0.631 | 1.004 (1.001-1.006) | 0.686 | -0.003 | 0.282 | 3.193(0.761-10.97) | 0.763 |
|  | Basophil count | 1.002 (0.997-1.006) | 0.080 | 1.001 (0.999-1.003) | **0.049** | 0.292 | 0.592 | 1.025(0.483-2.175) | 0.966 |
