# Supplementary figures and images for: A causal link between circulating leukocytes and three major urologic cancers: a mendelian randomization investigation
Source: Front Genet. 2024 Jun 19;15:1424119. doi: 10.3389/fgene.2024.1424119 (PMC11220253; doi:10.3389/fgene.2024.1424119)

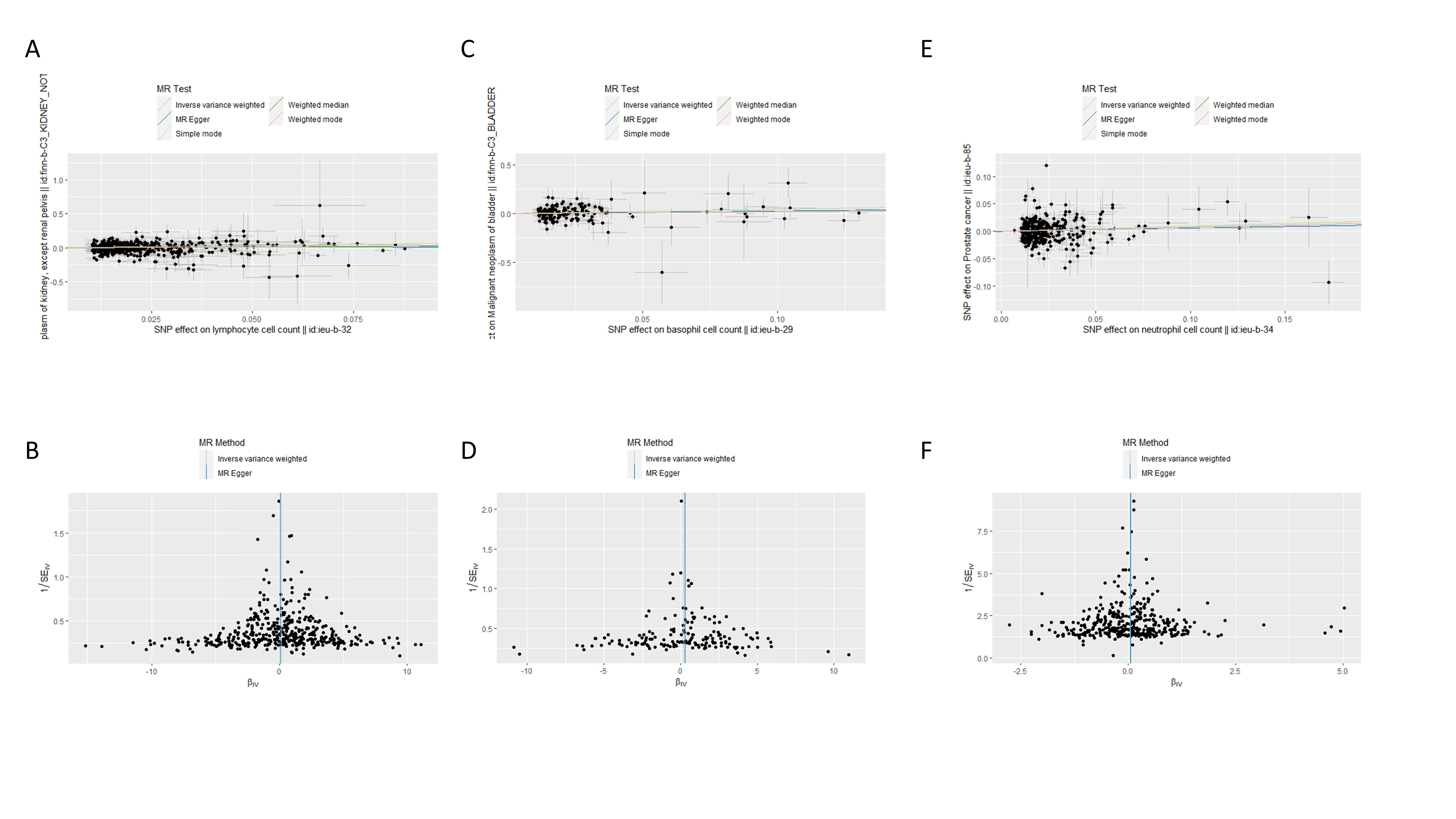

Supplement: Supplementary file 3 [file Image1.jpg]
