# Supplementary material for: A causal link between circulating leukocytes and three major urologic cancers: a mendelian randomization investigation
Source: Front Genet. 2024 Jun 19;15:1424119. doi: 10.3389/fgene.2024.1424119 (PMC11220253; doi:10.3389/fgene.2024.1424119)
Supplement: Supplementary file 4 [file Table2.doc]

**Table S2. Single-nucleotide polymorphisms (SNPs) used as instruments for differential leukocyte counts**

| **Phenotype** | **Number of SNPs Identified in GWAS (P < 5 × 10^−8^)** | **Fraction of variance explained by SNPs** | **F-tatistic** |
| --- | --- | --- | --- |
| Lymphocyte count | 422 | 7.71% | 131.098 |
| Monocyte count | 421 | 8.76% | 169.097 |
| Neutrophil count | 354 | 5.91% | 118.485 |
| Eosinophil count | 375 | 7.91% | 160.253 |
| Basophil count | 162 | 2.57% | 108.680 |
